# Development and Validation of a Liquid Chromatography–Tandem Mass Spectrometry Method for Screening Potential Citrate Lyase Inhibitors from a Library of Marine Compounds

**DOI:** 10.3390/md22060245

**Published:** 2024-05-27

**Authors:** Jiahong Wang, Huashi Guan, Zhe Xu

**Affiliations:** 1 Key Laboratory of Marine Drugs, Chinese Ministry of Education, Shandong Provincial Key Laboratory of Glycoscience and Glycoengineering, School of Medicine and Pharmacy, Ocean University of China, Qingdao 266003, China; 17861406010@163.com (J.W.); hsguan@ouc.edu.cn (H.G.); 2 Laboratory for Marine Drugs and Bioproducts, Innovation Center for Marine Drugs Screening and Evaluation, Qingdao National Laboratory for Marine Science and Technology, Qingdao 266237, China; 3 Marine Biomedical Research Institute of Qingdao, Qingdao 266071, China

**Keywords:** citrate lyase inhibitors, phenolic compounds, terpenoids compounds, UHPLC-QTOF MS, tuberculosis

## Abstract

Tuberculosis, a persistent illness caused by *Mycobacterium tuberculosis*, remains a significant global public health challenge. The widespread use of anti-tuberculosis drugs has resulted in the emergence of drug-resistant strains, which complicates treatment efforts. Addressing this issue is crucial and hinges on the development of new drugs that can effectively target the disease. This involves identifying novel therapeutic targets that can disrupt the bacterium’s survival mechanisms in various environments such as granulomas and lesions. Citrate lyase, essential for the survival of *Mycobacterium* species at lesion sites and in granulomatous conditions, is a potential target for the treatment of tuberculosis. This manuscript aimed to construct an efficient enzyme inhibitor screening platform using ultra-high performance liquid chromatography-quadrupole-time-of-flight mass spectrometry (UHPLC-QTOF MS). This system can accurately identify compounds with enzyme inhibitory activity from a library of marine terpenoids and phenolic compounds. Utilizing the screened herbal enzyme inhibitors as a starting point, we analyzed their chemical structures and skillfully built a library of marine compounds based on these structures. The results showed that all of the tested compounds from the phenolics library inhibited citrate lyase by more than 50%, and a significant portion of terpenoids also demonstrated inhibition, with these active terpenoids comprising over half of the terpenoids tested. The study underscores the potential of marine-derived phenolic and terpenoid compounds as potent inhibitors of citrate lyase, indicating a promising direction for future investigations in treating tuberculosis and associated disorders.

## 1. Introduction

Tuberculosis (TB) is a chronic and intricate illness stemming from infection with the slow-growing bacterium *Mycobacterium tuberculosis* [1]. It is primarily airborne and enters the body via the respiratory tract. *Mycobacterium tuberculosis* enters the body and initiates an intricate sequence of pathophysiologic reactions that ultimately result in the development of tuberculosis. Tuberculosis, a significant contributor to the worldwide pandemic, was the most lethal infectious illness in the world prior to the emergence of the new coronavirus infection. In November 2023, the World Health Organization (WHO) released information that 1.3 million people (167,000 people also infected with HIV) died of tuberculosis in 2022.

*Mycobacterium tuberculosis* demonstrates remarkable resilience in harsh environments including anaerobic and carbon dioxide-rich conditions in diseased tissues and granulomas. This resilience originates from its ability to adapt to hypoxic settings, maintaining survival by switching its metabolic pathway from the tricarboxylic acid (TCA) cycle to the reductive tricarboxylic acid (rTCA) cycle. Furthermore, *Mycobacterium tuberculosis* utilizes the rTCA cycle to consume carbon dioxide, maintaining both the carbon cycle and pH levels essential for growth [2]. Citrate lyase, a crucial enzyme in the rTCA cycle [3], catalyzes the conversion of citric acid to oxaloacetic acid. Citrate lyase promotes carbon fixation, which restores the balance between oxidative and reductive reactions during environmental transformations, thus allowing *Mycobacterium tuberculosis* to survive, grow, and survive in granulomas [2]. The pathogenesis of *Mycobacterium tuberculosis* relies significantly on citrate lyase [4]. Taken together, citrate lyase is a potential drug target for tuberculosis treatment.

Studies on citrate lyase inhibitors are limited. However, literature reviews have revealed substantial structural and functional similarities between ATP citrate lyase and citrate lyase, suggesting that insights from ATP-citrate lyase inhibitors can be leveraged to explore citrate lyase inhibitors. Both of them are present in the tricarboxylic acid cycle, exhibiting similar reaction mechanisms: ATP citrate lyase converts citric acid and coenzyme A to oxaloacetic acid and acetyl coenzyme A, while citrate lyase converts citric acid to oxaloacetic acid [5]. Notably, the γ-subunits of citrate lyase and ATP citrate lyase can interchangeably facilitate these enzymatic processes [6]. Furthermore, both enzymes involve the β-subunit, which converts citric acid to oxaloacetic acid [7,8] and displays cofactor cross-reactivity [9]. Their catalysis exhibits remarkable stereospecificity, with both enzymes producing C2 and C4 fragments from citric acid through a reversible aldol condensation-type reaction [10]. Given these parallels, it was hypothesized that ATP-citrate lyase inhibitors might inhibit citrate lyase activity. It is feasible to incorporate known ATP-citrate lyase inhibitors into the construction of compound libraries to screen citrate lyase inhibitors. This strategy will likely enhance the screening’s success and efficiency, accelerating the research process.

Enzyme activity quantifies the catalytic impact of an enzyme on a chemical reaction. Currently, inhibitor development depends greatly on precise enzyme activity measurements. The primary methods for determining enzyme activity are spectroscopy [11], chromatography [12], and mass spectrometry [13]. In particular, liquid chromatography-mass spectrometry (LC-MS) merges the benefits of liquid chromatography for complex sample separation with the selectivity and sensitivity of mass spectrometry, offering robust support for enzyme activity research. The recent focus on quantifying enzyme product content using LC-MS in enzyme inhibitor studies highlights its growing importance and potential in this research area [14,15,16,17,18,19].

*Salvia miltiorrhiza*, *Rhubarb*, *Lycium barbarum*, *Latycodon grandiflorum*, and *Rosa laevigata Michx* are herbs with antibacterial properties. Specifically, protocatechuic aldehyde and salvinorin B from *Salvia miltiorrhiza* hinder Gram-negative bacteria such as *Pseudomonas aeruginosa* [20]. Its fat-soluble components like essential oils show antimicrobial activity against various bacteria [21]. Rhubarb’s anthraquinones and water-soluble constituents exhibit antibacterial activities with multiple bacterial strains [22,23]. Lycium barbarum’s constituents, primarily water-soluble, have antibacterial properties. For instance, (+)-Lyoniresinol-3-O-β-glucoside, a glycoside, inhibits methicillin-resistant *Staphylococcus aureus* [24] while flavonoids are bacteriostatic against Gram-negative bacteria [25]. Flavonoids from *Platycodon grandiflorum* can be antibacterial [26]. In *Rosa laevigata Michx*, glycosides show moderate antifungal activity against *Candida albicans* and *Candida krusei* in vitro [27]. Additionally, terpenoids, the fat-soluble constituents of *Rosa laevigata Michx*, inhibit the effects of *Staphylococcus aureus*, *Bacillus cereus*, and *Escherichia coli* [28]. With their diverse antimicrobial mechanisms, these herbs present a promising natural alternative for developing therapeutic agents.

Marine environments are the largest aquatic ecosystems in the world and are also the most important source of organisms [29]. Marine organisms are known to produce various secondary metabolites with unique chemical structures [30]. These compounds of marine organisms have significant biological activities including anticancer, anti-inflammatory, antiviral, and antibacterial [31]. Additionally, the success rate of marine natural products is four times higher than that of other naturally derived compounds in drug discovery [32]. We utilized the marine compound library to screen for enzyme inhibitors to improve the success rate.

This study aimed to establish an efficient and reliable method for enzymatic studies and the inhibitor screening of citrate lyase using UHPLC-QTOF MS. The method has undergone rigorous validation by the Bioanalytical Method Validation Guidelines issued by the U.S. Food and Drug Administration covering selectivity, linearity, precision, accuracy, stability, and matrix effects. The five herbs known for their inhibitory effects were selected, and their active components were analyzed for citrate lyase inhibition. Based on the structures of these active ingredients and the functionally similar ATP-citrate lyase inhibitors, a specialized marine compound library was constructed. The efficacy of this library was assessed by measuring the inhibition rates against citrate lyase. In addition, not only is it the first work to establish a system for screening citrate lyase inhibitory activity inhibitors, but it also utilized UHPLC-QTOF MS for the more efficient and precise screening of inhibitors derived from the marine compound library by quantifying the product oxaloacetic acid. This study will provide an important scientific basis for screening citrate lyase inhibitors and promote the further innovation and development of possible drugs or pharmacophores for the treatment of tuberculosis.

## 2. Results

### 2.1. Optimization of UHPLC-QTOF MS Conditions

**Figure 1:**
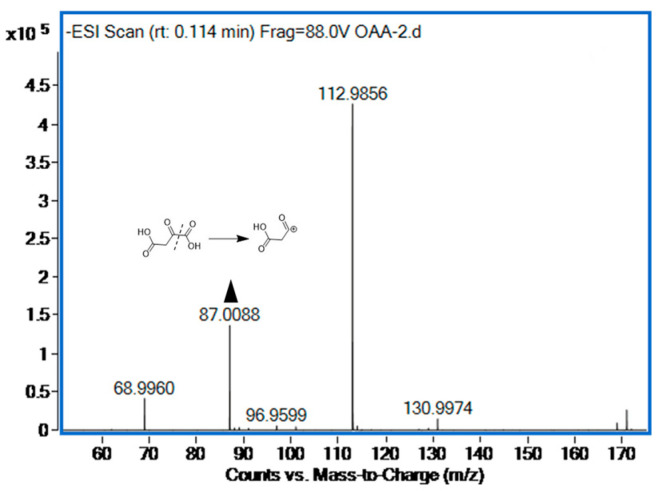
The oxaloacetic acid (500 µg/mL) mass spectra of negative mode.

The oxaloacetic acid was quantified by ultra-high performance liquid chromatography-quadrupole-time-of-flight mass spectrometry. The compounds’ inhibition of citrate lyase was assessed by measuring the reduction in oxaloacetic acid production. Oxaloacetic acid, which is both an α-keto and β-keto acid, rapidly decarboxylates due to the instability of its β-keto form. We found that the analysis of pure oxaloacetic acid revealed a significantly higher ion peak at 87.0088 ([M-COOH]^−^) compared to 130.9986 ([M-H]^−^). Consequently, our optimization efforts concentrated on the *m*/*z* value of 87.0088 (Figure 1).

The liquid chromatography and mass spectrometry conditions were optimized to enhance the response intensity of the enzymatic product oxaloacetic acid in the enzymatic reaction solution. A range of chromatographic conditions was investigated including mobile phases (acetonitrile, water, and methanol), additives (ammonium acetate and formic acid), and so on, in order to achieve sufficient chromatographic performance. Oxaloacetic acid (*m*/*z* 87.0088) was analyzed using an ACQUITY UPLC BEH C18 column. The mobile phases comprised 90% water with 0.1% *v*/*v* formic acid (Phase A) and 10% acetonitrile (Phase B).

The detection of the enzyme reaction products were optimized in both positive and negative ion modes by a full-scan mass spectrometry method. The results showed that the product oxaloacetic acid achieved a higher-intensity signal in the negative mode. The fragmentor voltage was found to be 88 V. The ion source parameters (capillary voltage, nebulizer, source temperature, and gas flow) were also optimized for a good analyte response. The detailed optimization findings are listed in Section 4.2.

### 2.2. Methodological Validation

The optimized UHPLC-QTOF MS method was validated for accuracy, precision, linearity, stability, and matrix effects according to the U.S. Food and Drug Administration Bioanalytical Method Validation Guidelines [33]. Specific data are supplemented in Appendix A.

#### 2.2.1. Selectivity

**Figure 2:**
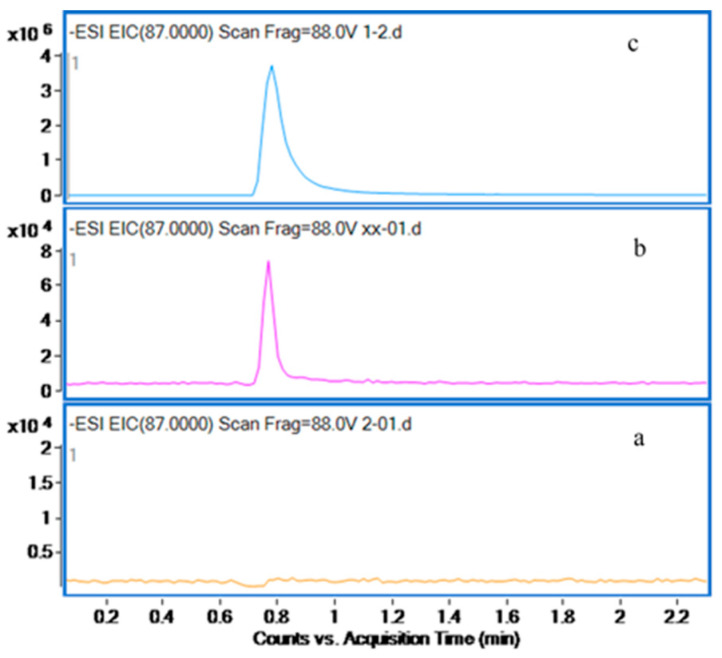
Extracted ion chromatograms of (**a**) the mixed solution of citric acid solution and Tris buffer solution, (**b**) the lowest point of the calibration curve, and (**c**) oxaloacetic acid in water (500 µg/mL).

Specificity experiments ensure that the experimental method accurately identifies the target analyte without non-target interference, thus safeguarding result accuracy and reliability. Specificity was assessed by separately analyzing the standard product solution and the mixed solution of citric acid and Tris buffer solution. Figure 2 shows that no product peaks were observed in the blank substrate solution, effectively differentiating between the substrate and product. This confirms the method’s reliability and accuracy.

#### 2.2.2. Linearity and the Limit of Detection

The aim of assessing linearity is to verify that the analytical method responds proportionally to varying concentrations of oxaloacetic acid, ensuring the method’s accuracy and reproducibility. Linearity was assessed using a seven-point calibration curve for oxaloacetic acid. The resulting linear regression equation was y = 8524.71x ± 2470.79, demonstrating linearity from 10 mg/L to 200 mg/L. The LOQ of oxaloacetic acid was 10 mg/L, the LOD of oxaloacetic acid was 4 mg/L, and the range of R^2^ was 0.9982–0.999 (Table A1 and Table A2). The results described above indicate that the quantitative method based on UHPLC-QTOF MS is reliable.

#### 2.2.3. Accuracy and Precision

Accuracy and precision can ensure the reliability and validity of the analytical method. Accuracy is defined as the relative error (RE, %) from the theoretical concentrations, while precision is expressed as the relative standard deviation (RSD, %) of the QC samples. The intraday (*n* = 6) and interday (*n* = 3) precision and accuracy were investigated using QC samples at four different concentration levels (10 mg/L, 20 mg/L, 50 mg/L, and 160 mg/L) for oxaloacetic acid. Intraday precision of the QC samples was 0.96–2.65%, with accuracies from 92.72 to 99.44%. Interday precision ranged from 6.52 to 11.23%, with 90.63–103.44% accuracy. The required accuracy range for the assay was 85–115%, and the precision range for the assay was ±15%. Therefore, the results fully met the acceptance criteria. The high accuracy and precision demonstrate the reliability of the proposed method for determining oxaloacetic acid in Tris buffer solution.

#### 2.2.4. Stability

The stability test was designed to ensure that the oxaloacetic acid remained stable under various conditions and confirm the analytical method’s reliability and accuracy. We assessed the stability of oxaloacetic acid in the buffer solution under both short-term and long-term storage (3 weeks at −20 °C). Short-term stability assessments included 24-h bench stability at room temperature, 24-h autosampler stability, and stability over three freeze–thaw cycles between room temperature and −20 °C within 48 h. The short-term stability studies showed accuracy levels for the LOQ and QC samples between 85.36 and 106.95% with a precision of 1.67–9.95%. Long-term stability showed accuracy levels from 88.41 to 101.73% and precision from 2.07 to 8.96%. These findings confirm the stability of oxaloacetic acid under the tested conditions as all of the observed values fell within the accepted accuracy and precision ranges.

#### 2.2.5. Matrix Effects

Matrix effect studies are conducted to evaluate the interference of Tris buffer solutions on analytical determinations, ensuring the accuracy and reliability of the methods. During the matrix effects study, oxaloacetic acid samples were prepared at the same concentration as the blank solvent (water), and the matrix effects were evaluated based on the recovery of standard oxaloacetic acid in buffer solution. Three concentration levels of 20, 50, and 160 mg/L were analyzed to determine the minimal ionic inhibition (<15%) for oxaloacetic acid. The results showed that the method for the quantification of oxaloacetic acid was not affected by the operating environment.

### 2.3. Enzyme Reaction

Citrate lyase catalyzes the cleavage of citric acid into oxaloacetic acid and acetic acid. This enzymatic reaction provides energy to the cell and is crucial for maintaining the intracellular acid–base balance and synthesizing organic acids. The efficiency and yield of enzyme reactions are influenced by various factors including the quantity of enzyme, the duration of the reaction, the composition of the reaction mixture, and the concentration of the substrate.

**Figure 3:**
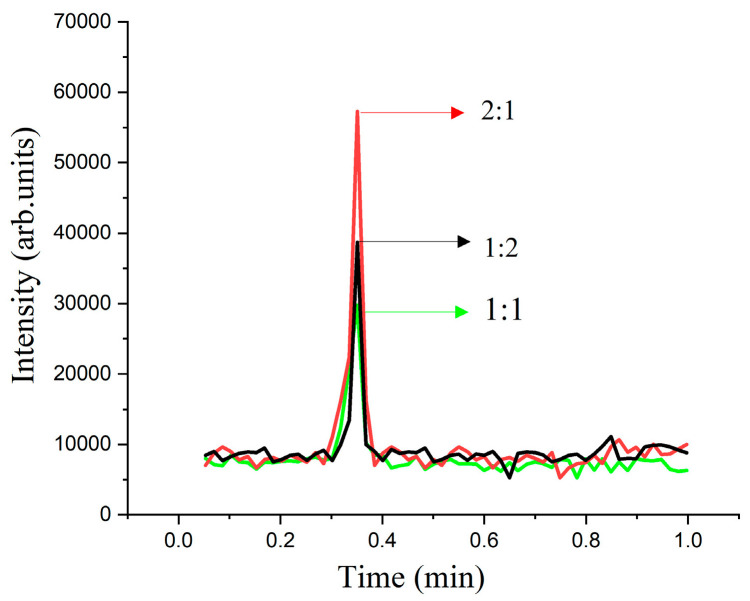
The effect of enzyme-to-substrate dosage ratio on the activity of citrate lyase. Under conditions of 10 µg/mL citric acid, 1 U/mL enzyme, 37 °C, and 30 min reaction time.

The enzyme dosage used directly influences the reaction rate. Increasing the enzyme levels accelerates the reaction by facilitating more enzyme–substrate interactions. At low enzyme dosages, the product oxaloacetic acid was undetectable. To investigate the impact of enzyme concentration on oxaloacetic acid production, we varied the ratio of citric acid to enzyme, keeping the volume and the enzyme concentration constant. The citrate concentration was maintained at a 10 µg/mL constant, and the volume was adjusted using deionized water. To study the effect of different ratios of citrate to enzyme (1:1, 1:2, and 2:1) on the production of oxaloacetic acid, the enzyme concentration was kept at 1 U/mL. It was discovered that the optimal ratio of citric acid to enzyme dosage for maximizing the production of oxaloacetic acid was 1:2 by comparing the experimental outcomes depicted in Figure 3. A quantity of 40 µL was identified as the optimal enzyme dosage.

**Figure 4:**
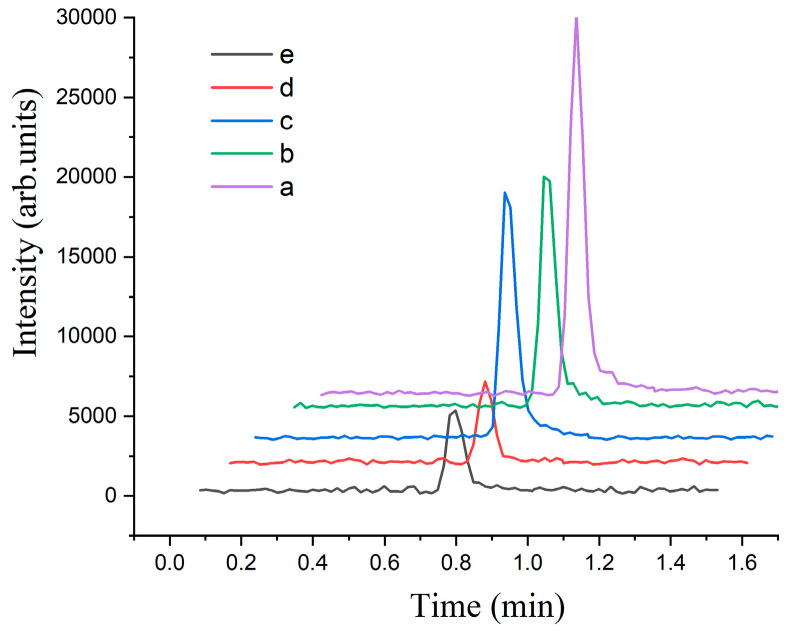
Effects of enzyme reaction components on the activity of citrate lyase after 30 min, with substrate citric acid concentration fixed at 100 mg/mL and reaction temperature at 37 °C. Experimental conditions: (a) control (without coenzyme A, ATP, and Mg^2+^); (b) with ATP; (c) with coenzyme A; (d) with coenzyme A and ATP; (e) with ATP, coenzyme A, and Mg^2+^.

The reaction components were optimized based on the previously determined optimal enzyme dosage. As Figure 4 illustrates, experiments b and c, which involved the addition of ATP solution and coenzyme A, respectively, exhibited smaller peak areas of oxaloacetic acid in comparison to the control solution a. Notably, the control solution did not contain ATP, coenzyme A, or Mg^2+^. This observation suggests that the introduction of coenzyme A and ATP may have decelerated the enzymatic activity. Specifically, ATP is known to interact with the β-subunit of citrate lyase, a crucial component for the cleavage process [2].

Citrate lyase catalyzes the conversion of citric acid into oxaloacetic acid, facilitated by Mg^2+^. To assess the influence of Mg^2+^, we compared the reaction intensity with and without its addition. It is worth mentioning that the solid enzyme preparations used in our experiments already contained Mg^2+^. The comparison between experiments e and d revealed that the inclusion of Mg^2+^ had a minimal impact on the enzymatic reaction. This finding suggests that the endogenous Mg^2+^ present in the enzyme preparations was sufficient to support the enzymatic activity, and additional Mg^2+^ did not significantly enhance or alter the reaction.

In summary, Figure 4 provides valuable insights into the effects of various reaction components on the enzymatic activity. The observed reductions were caused by coenzyme A and ATP as well as the minimal impact of Mg^2+^.

**Figure 5:**
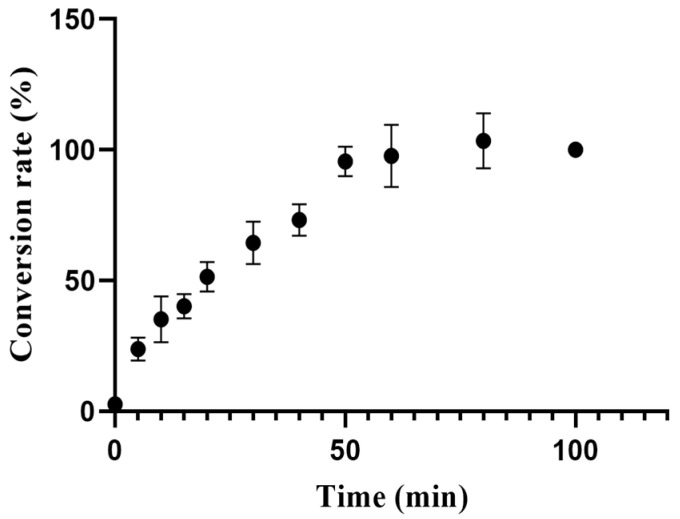
The impact of enzyme reaction time on the generation of the enzyme product, oxaloacetic acid, was investigated while maintaining a fixed concentration of substrate citric acid at 100 mg/mL and a reaction temperature of 37 °C. Each data point was replicated three times.

The enzyme reaction time was optimized based on the previously determined optimal enzyme dosage and reaction components. Specific time points were set including 0 min, 5 min, 15 min, 20 min, 30 min, 40 min, 50 min, 60 min, and 80 min. All other parameters were held constant. Starting from 50 min, the peak area of oxaloacetic acid remained stable, as shown in Figure 5. This suggests that the production of oxaloacetic acid was approaching equilibrium. After a reaction time of 50 min, the production of oxaloacetic acid remained consistent. However, the costs associated with maintaining the reaction conditions may increase. Therefore, 50 min was identified as the optimal reaction time, considering the product yield, reaction efficiency, and cost-effectiveness.

**Figure 6:**
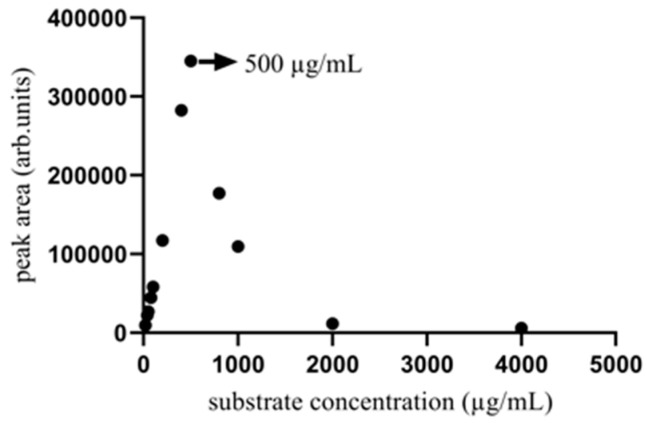
The effect of citric acid concentration on the oxaloacetic acid production of the enzyme product after 50 min when the enzyme reaction temperature was fixed at 37 °C. The concentration of citric acid that achieves the highest oxaloacetic acid yield exists.

Based on the previously optimized enzyme dosage, reaction components, and duration, a range of twelve concentrations of citric acid, spanning from 20 μg/mL to 4000 μg/mL, were tested within the enzymatic reaction system. The oxaloacetic acid ion peak was detected at an enzyme dosage of 40 µL, which was then maintained during the optimization of the substrate concentration. The concentration of citric acid significantly influenced the enzymatic reaction within a given timeframe, as demonstrated in Figure 6. An increase in substrate concentration, particularly from 0 to 500 μg/mL of citric acid, led to more collisions of the enzyme–substrate. More substrate molecules can bind to the enzyme, leading to the increased production of oxaloacetic acid. However, the reaction rate decreased when the citric acid concentration was higher than 500 μg/mL. This decrease may be due to the saturation of the enzyme’s active site by an excessive amount of substrate molecules or the inhibitory effect of a high substrate concentration on the reaction efficiency. The maximum oxaloacetic acid production, as shown in Figure 6, occurred at a substrate concentration of 500 µg/mL. Consequently, a 500 µg/mL concentration of citric acid was selected as optimal to maximize the enzymatic reaction effectiveness and minimize the risks associated with excessive substrate concentrations such as wastage or inhibition.

The optimal conditions for the enzyme reaction system were established by integrating previous optimization results. The reaction system configuration was as follows: 40 µL of deionized water was mixed with 110 µL of 20 mM Tris buffer. Subsequently, 80 µL of citric acid solution at a concentration of 500 µg/mL was added to this mixture. Next, 40 µL of the enzyme solution was mixed into the solution. Finally, the enzyme reaction system was incubated in a 37 °C water bath for 50 min to enable the enzyme-catalyzed reaction.

### 2.4. Determination of Enzyme Inhibitory Components in Herbs

#### 2.4.1. Natural Inhibitors of Citrate Lyase

The five herb powders, consisting of *Salvia miltiorrhiza*, *Rhubarb*, *Lycium barbarum*, *Latycodon grandiflorum*, and *Rosa laevigata Michx*, were extracted using 75% (*v*/*v*) methanol, a typical solvent for this process. The negative control was 75% (*v*/*v*) methanol. *Lycium barbarum*, *Latycodon grandiflorum*, and *Rosa laevigata Michx* were found to promote citrate lyase activity. *Rhubarb* demonstrated significant citrate lyase inhibition, with a rate of 39.57% compared to the negative control of 75% (*v*/*v*) methanol. *Salvia miltiorrhiza* exhibited a 15.69% inhibition rate. Consequently, *Salvia miltiorrhiza* and *Rhubarb* were identified as herbal sources containing potential enzyme inhibitors for citrate lyase.

In order to eliminate the potential impact of regional variations and differences in herb processing on the results, *Salvia miltiorrhiza* and *Rhubarb* were sourced from three locations: Rongde Ankang Clinic in Lichang District, Ankang Pharmacy in Pingdu District, and Beijing Tongren Housing in Laoshan Distric. *Rhubarb* was processed with water, and *Salvia miltiorrhiza* was extracted with a 20% methanol solution. Results from all three regions demonstrated enzyme inhibitory effects. The findings reconfirmed the inhibitory properties of *Salvia miltiorrhiza* and *Rhubarb* against citrate lyase.

#### 2.4.2. Identification of Inhibitory Components of *Rhubarb* and *Salvia miltiorrhiza*

(1) Identification of inhibitory components of *Salvia miltiorrhiza*

**Figure 7:**
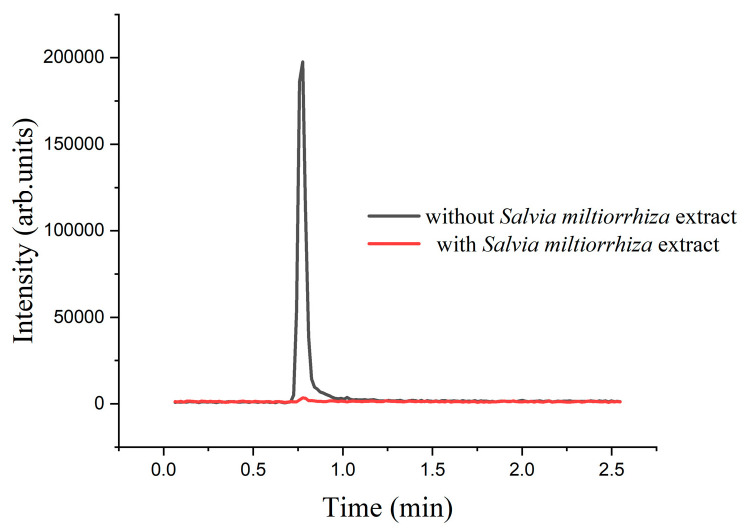
Chromatograms with or without the addition of *Salvia miltiorrhiza* extracts.

Section 2.4.1 indicates that *Salvia miltiorrhiza’s* primary enzyme-inhibiting components are water-soluble. According to literature reports, the water-soluble components of *Salvia miltiorrhiza* include salvianolic acids, salvinorin, salvinorin A, protocatechuic aldehyde, salvinorin B, and perillic acid [34]. Following the addition of a *Salvia miltiorrhiza* aqueous extract to the enzyme reaction solution, no oxaloacetic acid ionic peak was detected, as shown in Figure 7. Among the phenolic acid components in *Salvia miltiorrhiza*, salvianolic acid B was the principal component and the most active among the water-soluble salvianolic substances [35]. Pure salvianolic acid B was dissolved in water to prepare a 10 mM solution. Adding the salvinorin B solution to the enzyme reaction resulted in inhibition rates of 91.36% and 91.89%, relative to the negative control (water). Therefore, salvianolic acid B was identified as the specific enzyme inhibitory component in *Salvia miltiorrhiza*.

(2) Identification of inhibitory components of *Rhubarb*

The inhibitory effect of water-soluble components of Rhubarb on citrate lyase was mainly studied. Rhubarb polysaccharides are primarily extracted and purified through water extraction and alcohol precipitation. These steps are detailed in Section 4.4. The negative control of rhubarb polysaccharide was water, which served as the solvent for the polysaccharide. A total of 10 mg of extracted rhubarb polysaccharide was dissolved in 1 mL of water. The inhibition of citrate lyase activity by rhubarb polysaccharides extracted by the two different extraction scheme was 22.60% and 23.11%, respectively, as compared to the negative control.

Rhubarb anthraquinone, a fat-soluble component, is regarded as the primary pharmacologically active compound in Rhubarb. Emodin officinale exhibits inhibitory activity against ATP-citrate lyase [36]. Thus, anthraquinones were employed to evaluate their potential inhibitory effect on citrate lyase in this experiment. Anthraquinones in Rhubarb include rhodopsin, rhubarbic acid, aloe barbadensis, rhodopsin methyl ether, and rhubarb phenol. The negative controls for anthraquinone were prepared with a 50% (*v*/*v*) ethanol solution. Rhubarb acid, compared to the negative control, promoted enzyme activity. In contrast, emodin exhibited a 68.92% inhibition rate. The addition of rhubarb acid-8-o-glucoside, rhubarb phenol, and rhubarb 6-methyl ether yielded an oxaloacetic acid peak area in the enzyme system that was similar to the negative control. The MassHunterB.04.00 software identified a −2.79 ppm discrepancy in molecular weight for emodin between the theoretical and assayed values after dehydrogenation. An error of 5 ppm in molecular mass indicates the accurate quantification of emodin by mass spectrometry.

These findings suggest that emodin and rheum polysaccharides in Rhubarb are potential enzyme inhibitors. These compounds share phenolic structures, which were used as the basis for screening. This discovery guides the development of marine compound libraries.

### 2.5. Screening Marine Libraries for Potential Enzyme Inhibitors

Marine compounds exhibit unique structural patterns compared to land-based organisms. These compounds often incorporate chloride and bromide ions, imparting unique structural and functional properties. These properties may yield unique pharmacological effects, potentially leading to the discovery of new enzyme inhibitors. Consequently, the utilization of marine chemical libraries for enzyme inhibitor screening could significantly advance drug discovery.

**Figure 8:**
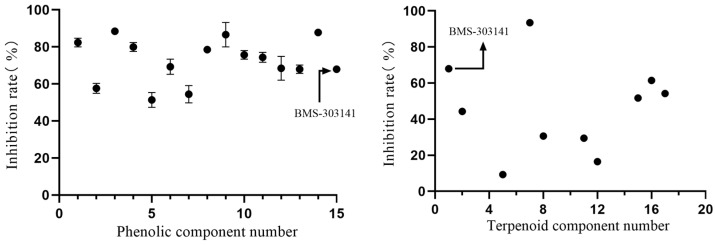
Plot depicting the impact of components from marine compound libraries on enzyme inhibition.

Section 2.4 reports that salvinorin B and emodin exhibit enzyme inhibitory properties. Based on these structures, the marine compound library encompassed a collection of phenolic marine compounds. Terpenoids were found to suppress ATP-citrate lyase [37]. Thus, the marine compound library comprises phenols, terpenoids, and their derivatives. All marine phenolic compounds in the marine compound library showed a more than 50% inhibition rate, as shown in Figure 8. Furthermore, nearly half of the terpenoids from the marine chemical library demonstrated efficacy against citrate lyase. The compounds were kept at a constant 10 mM concentration to enhance the inhibitory impact assessment. This mixing of terpenoids led to significant inaccuracies in individual data points. Therefore, averages were calculated to provide a comprehensive analysis of the experimental results.

### 2.6. Kinetic Studies of Enzyme Reactions

**Figure 9:**
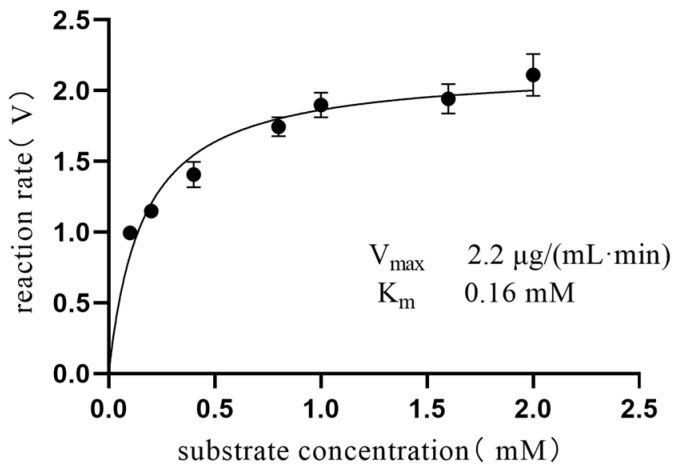
The Michaelis–Menten plot of citrate lyase. The reaction rate for citrate lyase was calculated by dividing the measured oxaloacetic acid concentration by a reaction time of 5 min. The concentration of oxaloacetic acid was determined using a linear calibration curve for oxaloacetic acid.

Enzymatic kinetics, crucial for studying enzyme–substrate interactions, forms the foundation of understanding the reaction mechanisms and pharmaceutical development. The Michaelis constant (K_m_) indicates the substrate concentration where the reaction rate is half its maximum, a key parameter in enzymatic kinetics. K_m_ uniquely depends on the enzyme type, unaffected by concentrations of either the enzyme or substrate. The K_m_ value was determined to be 0.16 mM, and the V_max_/K_m_ ratio was calculated to be 13.49, as illustrated in Figure 9. These values were obtained by analyzing the data using the Michaelis–Menten model. Comparison of the obtained data with the literature values (0.14 mM [38]) revealed a high level of concordance. Overall, the alignment of the experimental results with the literature values validates the reliability and precision of the methodology used.

**Figure 10:**
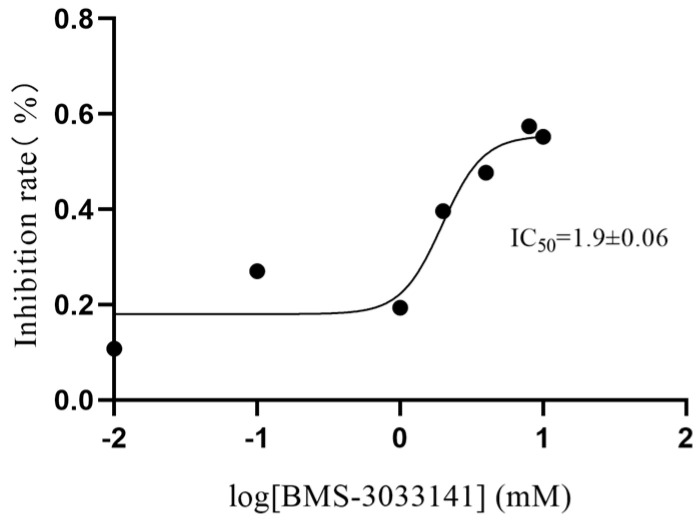
Concentration–dependent inhibition curve of citrate lyase by BMS-303141.

BMS-303141, a potent and cell-permeable chemical compound, has emerged as a positive inhibitor of ATP-citrate lyase [39]. The IC_50_ value of BMS-303141, as an inhibitor of citrate lyase, was determined using an enzyme inhibition assay. The assay involved quantifying the inhibitory impact of BMS-303141 on the citrate lyase activity across various concentrations. The inhibition rate was determined using Equation (1), as outlined in Section 4.7. To further enhance the accuracy of quantifying this inhibitory effect, a curve-fitting approach was employed to graphically represent the relationship between the drug concentration and enzyme activity. The half inhibitory concentration (IC_50_) of BMS-303141 was calculated to be 1.9 mM, as depicted in Figure 10. This outcome provides a vivid illustration of BMS-303141’s inhibitory potency against citrate lyase.

## 3. Discussion

This study makes strides in identifying marine-derived phenolic and terpenoid compounds as potent inhibitors of citrate lyase, crucial for *Mycobacterium tuberculosis* survival and potentially instrumental in TB treatment. This discovery aligns with the growing recognition of the unique biochemical properties of marine compounds and their potential for drug discovery.

Citrate lyase is currently found only in prokaryotes including *Klebsiella pneumoniae*, *Mycobacterium tuberculosis*, and *Rhodopseudomonas* [9]. Citrate lyase plays a crucial role in the survival of mycobacteria under anaerobic conditions. Hu et al. found that knocking down the gene for the β-subunit of citrate lyase in *Mycobacterium bovis* BCG led to reduced survival under low-oxygen conditions [2]. Garima et al. found that the β-subunit of citrate lyase is essential for the pathogenesis of *Mycobacterium tuberculosis*, as evidenced by mutant strains [4]. The β-subunit of *Klebsiella pneumoniae* citrate lyase complexes is homologous to *Mycobacterium tuberculosis* [2]. Therefore, the citrate lyase of *Klebsiella pneumoniae* could be a suitable agent for initial screening. The β-subunit is responsible for oxaloacetic acid production. Consequently, this study aimed to preliminarily assess the effects of potential inhibitors on the citrate lyase β-subunit, measured by oxaloacetic acid production.

Research has revealed significant similarities in the function and structure of citrate lyase and ATP-citrate lyase [5,6,7,8,10,40]. Therefore, we proposed the bold hypothesis that compounds inhibiting ATP-citrate lyase could also suppress the citrate lyase activity. We deliberately included known ATP-citrate lyase inhibitors in the compound library to aid in discovering citrate lyase inhibitors. For example, Koerner et al. found that emodin inhibits the ATP-citrate lyase [36]. Thus, we emphasized the extraction and isolation of rhubarb anthraquinones during the active constituent extraction process from *Rhubarb*. Our study found that emodin significantly reduced citrate lyase’s production of oxaloacetic acid. Zhan et al. reported that BMS-303141 inhibits ATP-citrate lyase [39]. Li et al. found that terpenoids also inhibit ATP-citrate lyase [37]. Additionally, our research identified sulfonamides (e.g., BMS-303141) and diterpenes (e.g., vitamin A) as inhibitors of citrate lyase. We found the novel result that citrate lyase and ATP-citrate lyase may have a close functional relationship.

Our findings found that the structure of inhibitors emerged as a critical factor in the successful screening of enzyme inhibitors from marine chemical libraries. Carbone et al.’s discovery that indolyl-7-aza indolyl triazine compounds act as inhibitors for the pyruvate dehydrogenase kinase (PDK) enzyme underscores the value of structural specificity in guiding the selection of compound libraries [41]. We developed a phenolic compound library, drawing on the structural insights of emodin from *Rhubarb* and salvianolic acid B from *Salvia miltiorrhiza*. Additionally, we compiled a terpenoid library recognizing the role of terpenoids in ATP-citrate lyase inhibition. Remarkably, our findings indicate that the marine compound library’s phenolic compounds significantly inhibited citrate lyase. At 10 mM, these phenolic compounds showed substantial inhibitory effects, with each surpassing 50% inhibition. Conversely, while terpenoids also inhibited the enzyme, their inhibition levels varied significantly. This technique markedly enhanced both the efficiency and accuracy of our screening process, illustrating the importance of combining targeted structural analysis with sophisticated analytical methods to uncover promising enzyme inhibitors.

## 4. Materials and Methods

### 4.1. Chemicals and Reagents

Citrate lyase (EC 4.1.3.6, 120 units) (catalog no. 10354074001) and formic acid (purity ≥ 99%) were purchased from Sigma-Aldrich Chemical Co., LLC. (St. Louis, MO, USA), and LC-MS grade acetonitrile and methanol were acquired from Merck (Darmstadt, Germany). Double distilled water was sourced from A.S. Watson Group Ltd. (Hong Kong, China), while Aladdin Chemical (Shanghai, China) supplied trimethylaminomethane, magnesium chloride hexahydrate, and dimethyl sulfoxide (DMSO). Anhydrous citric acid (purity ≥ 99%) was provided by InnoKai Technology Co., Ltd. (Beijing, China). The tuning fluid and reference fluid were provided by Agilent Technology Co., Ltd. (Beijing, China).

Oxaloacetic acid (purity ≥ 98%) was purchased from Mclin Medical Equipment Products Co., Ltd. (Shanghai, China). BMS-303141 (purity ≥ 99.15%) was purchased from Bide Pharmatech Ltd. (Shanghai, China). Other inhibitors were purchased from Aladdin Global Corporation Limited, Shanghai Topscience Co., Ltd. (Shanghai, China) and Mclin Medical Equipment Products Co., Ltd. (Shanghai, China). Specific information is supplied in Appendix C.

### 4.2. UHPLC-QTOF MS Conditions

The analysis of CL was performed using an Agilent technologies 1290 Infinity II UHPLC system and 6530 Accurate-Mass Q-TOF UHPLC-ESI-MS (Santa Clara, CA, USA). The Agilent 1290 system consists of a G7117A DAD FS detector, a G7167B auto-sampler, a G7116B MCT column oven, and a G7120A high-speed pump.

For the UHPLC analysis, a Waters ACQUITY UPLC BEH C18 column (100 mm × 2.1 mm, 1.7 μm particle size) was used to separate the analytes. Solvent A was 0.1% (*v*/*v*) formic acid in water, and solvent B was CH_3_CN. The isocratic elution method consisted of a 0.4 mL/min flow rate, 90% formic acid in water, and 10% acetonitrile. Each sample was analyzed by 3 min of stoptime and 3 min of post-time. The sample injection volume was 2 uL. The column oven was set to 25 °C.

The mass spectrometer was operated in full MS scan mode with negative ionization electrospray. The detection conditions were as follows: capillary voltage: 3.5 kV; drying gas temperature: 350 °C; drying gas flow: 8 L/min; nebulization pressure: 30 psi; fragmentor: 88 V; skimmer: 65 V; Oct 1 RF Vpp: 750 V; calibration frequency: once a day. Centroid mass data within the mass range 50–1000 *m*/*z* were acquired with 1 spectra/s. The tuning fluid part number is G1969-85000, and the reference fluid part number is G1969-85001. The error obtained when calibrating has been added in Appendix D. Data analysis was conducted by Agilent Mass Hunter Qualitative Analysis software and the workstation (version B.08.00).

### 4.3. Sample Preparation

All herbal samples were crushed to a powder state in a pulverizer and filtered through a 40-mesh (0.45 mm) sieve to completely extract the active substances. The crushed herbs were weighed using a New Classic MF MS105DU electronic balance (Toledo, Switzerland) with a precision of 0.01 mg.

### 4.4. Extraction Procedure

The phenolic acids of *Salvia miltiorrhiza* were isolated through a series of steps: 1 g of powder was soaked in 100 mL of water, then filtered in an oil bath at 104 °C for 40 min. The filtrate was acidified to pH 2 with hydrochloric acid and then extracted with 50 mL of ethyl acetate. For the control, 100 mL of water was boiled for 40 min, adjusted to pH 2 using hydrochloric acid, and extracted with ethyl acetate. The ethyl acetate was evaporated, redissolved in 50 mL of water, and evaporated again after extraction. This process was repeated twice. The enzymatic reaction was analyzed with mass spectrometry.

Rheum polysaccharides were extracted using traditional water decoction and alcohol precipitation methods. Scheme 1 involved soaking 1 g of *Rhubarb* powder in 95% ethanol overnight, filtering it, and then boiling the filtrate with 10 mL of hot water (95 °C) for 30 min. After filtration, 20 mL of water was added to the filtrate, boiled, and the two filtrates were combined. The combined filtrate was washed with anhydrous ethanol and ether and then evaporated to yield rheum polysaccharides (test article 1). Scheme 2 involved mixing 2.5 g of *Rhubarb* powder with 75 mL of water, heating in a 95 °C water bath for 140 min, concentrating the mixture to 10× with 80% methanol, and leaving it overnight. The mixture was then centrifuged (1200 r/min for 20 min), the filtrate was washed with anhydrous ethanol and ether, and evaporated to dryness to obtain rhubarb polysaccharide test article 2.

Rhubarb anthraquinones were isolated through a series of steps: 50 g of *Rhubarb* powder and 100 mL of 95% ethanol were combined in a 250 mL round-bottom flask and refluxed for 200 min. The mixture was then filtered while still hot, and the residue was re-extracted by refluxing in a 95% ethanol bath. The combined filtrates were then concentrated to obtain an extract. The extract underwent liquid–liquid extraction using water, 150 mL of ether, and a 250 mL separatory funnel. After shaking for 20 min, the ether layer was separated, and this step was repeated five times. The combined ether solutions were collected. The aqueous layer was sampled (test article 1), as was the ether layer (test article 2). The ether layer underwent further treatment with 20 mL of 5% sodium bicarbonate in a separatory funnel, followed by extraction. The resulting lower liquid layer was collected and acidified with hydrochloric acid to achieve a pH of 2.39. After suction filtration, the mixture yielded a solid powder, identified as rheinic acid (test article 3).

The ether layer was moved to a 250 mL separatory funnel, to which 20 mL of 5% sodium carbonate was added. The mixture was shaken and allowed to settle, and the lower liquid layer was collected. This extraction step was repeated five times, each with 20 mL of sodium bicarbonate added. The pH was adjusted to about 2 with hydrochloric acid after combining the lower liquid layers. Filtration followed, collecting the solid powder (test article 4—emodin) that was then washed with water. The remaining ether layer was treated with 15 mL of 0.5% sodium hydroxide in the separatory funnel, then shaken and allowed to settle. The procedure was repeated three times, collecting the lower liquid layer after each addition of 15 mL of 0.5% sodium hydroxide. The ether layer underwent two water washes, the lower liquid layers were consolidated, and the pH was adjusted to about 2 with hydrochloric acid. The mixture was filtered to collect the solid powder (test article 5—aloe vera emodin), then washed and dried. The ether layer was processed further and evaporated to dryness, yielding test article 6 (containing chrysophanol and emodin monomethyl ether). The negative control of rhubarb polysaccharide was water, which served as the solvent for the polysaccharide. Test substances 3–6 were dissolved in 5 mL of 50% ethanol, using this solvent as the negative control.

### 4.5. Establishment of Enzyme Reaction System

This experiment aimed to assess the enzyme dosage that affects the citrate lyase reaction. The solution comprised 8 μL of 100 mM MgCl_2_, 40 μL of 2.64 mg/mL ATP, and 110 μL of 20 mM Tris buffer. Deionized water ensured a consistent total volume of the enzyme reaction solution. Initially, 20 μL of a citric acid solution with a concentration of 10 μg/mL was added to the mixture. Subsequently, enzyme solutions of 20 μL and 40 μL (1 U/mL each) were introduced. The reaction mixtures were placed in water incubated at 37 °C for 30 min. A sample of 120 μL from each reaction was mixed with 480 μL of acetonitrile. The mixture was centrifuged at 1200 r/min for 10 min. The 200 μL supernatant was filtered through a 0.22 µm membrane for analysis. The third experimental procedure replicated the first, with the sole variation being the citric acid volume, adjusted to 40 μL at a 10 μg/mL concentration.

This experiment assessed the various components affecting the citrate lyase reaction. Solution a involved 40 μL of deionized water, 110 μL of 20 mM Tris buffer, and 80 μL of 100 mg/mL citric acid. Solution b was prepared by adding 40 μL of 4 mg/mL of coenzyme A to solution a. Solution c was prepared by adding 40 μL of 2.64 mg/mL ATP to solution a. Solution d was prepared by adding 40 μL each of coenzyme A and ATP to solution a. Solution e was prepared by adding 4 μL of 100 mM Mg^2+^ to solution d. Afterward, 40 μL of a 5 mg/mL enzyme solution was introduced into each solution (a, b, c, d, and e). Each reaction mixture was incubated at 37 °C for 30 min. At the end of the reaction, 120 μL of each enzyme solution was mixed with 480 μL of acetonitrile and centrifuged at 1200 r/min for 10 min. Subsequently, 200 μL of the supernatant was filtered through a 0.22-μm membrane. The filtered samples were analyzed using UHPLC-QTOF MS to identify the enzymatic reaction products.

This experiment evaluated the effect of various reaction times on citrate lyase activity. The reaction solution was prepared by mixing 40 μL deionized water, 110 μL 20 mM Tris buffer, and 80 μL 100 mg/mL citric acid. A total of 40 μL of a 5 mg/mL enzyme solution was added to the mixture. Incubation occurred at 37 °C for durations of 0, 5, 15, 20, 30, 40, 50, and 60 min. The 120 μL of the enzyme reaction solution was combined with 480 μL of acetonitrile after each incubation. The mixture was centrifuged at 1200 r/min for 10 min. Subsequently, 200 μL of the supernatant was filtered through a 0.22-μm membrane. The filtered samples were analyzed with UHPLC-QTOF MS.

The experiment aimed to assess the effect of varying citric acid concentrations on citrate lyase activity. Solutions of citric acid were prepared at twelve concentrations including 20 μg/mL, 40 μg/mL, 50 μg/mL, 80 μg/mL, 100 μg/mL, 200 μg/mL, 400 μg/mL, 500 μg/mL, 800 μg/mL, 1000 μg/mL, 2000 μg/mL, and 4000 μg/mL. A total of 80 μL of the citric acid solution for each concentration was mixed with 110 μL of Tris buffer solution and 40 μL of water. Then, 40 μL of enzyme solution was added to the mixture. Reactions were incubated at 37 °C for 50 min. Afterward, 120 μL of the reaction solution was mixed with 480 μL of acetonitrile and centrifuged at 1200 r/min for 10 min. Finally, 200 μL of the supernatant was filtered through a 0.22 µm membrane for UHPLC-QTOF MS analysis to assess the enzymatic activity across the substrate concentrations.

### 4.6. Enzyme Assay and Kinetic Measurements

K_m_ values for citrate lyase were determined using a range of substrate concentrations. In order to study the enzyme kinetics, the incubation time was set at 5 min to ensure that the reaction rate was in the zero-order reaction stage. First, the enzyme was incubated with substrate concentrations ranging from 0.0052 to 52 mM for 5 min. Significantly, insufficient substrate concentrations yielded no product, while excessive ones decreased product formation. A concentration range of 0.01–2.08 mM was selected for further analysis.

Citric acid solutions were prepared at concentrations of 0.1 mM, 0.2 mM, 0.4 mM, 0.8 mM, 1.0 mM, 1.6 mM, and 2 mM. A total of 80 µL of the citric acid solution for each concentration was mixed with 110 µL of Tris buffer solution and 40 µL of water. Subsequently, 40 µL of enzyme solution was added to the mixture and incubated at 37 °C for 5 min to start the reaction. Afterward, 120 μL of the reaction solution was mixed with 480 μL of acetonitrile and centrifuged at 1200 r/min for 10 min. Then, 200 µL of the supernatant was filtered through a 0.22-µm membrane for UHPLC-QTOF MS analysis to assess the enzymatic activity across the substrate concentrations. Measured data were used to correlate the substrate concentration with enzymatic activity and plot Michaelis–Menten curves, visually depicting their relationship. These curves derived key kinetic parameters such as K_m_ (Michaelis constant) and V_max_ (maximum reaction rate).

### 4.7. Inhibition Study

The reaction system was initiated by mixing 80 μL of the citric acid solution with 110 μL of Tris buffer solution, then adding 40 μL of each test article and enzyme solution. The mixture was incubated at 37 °C for 50 min. Equation (1) presents the formula to calculate the inhibition rate [42]:

(A_0_ − A_i_)/A_0_ × 100% = inhibition (%) (1)

A_i_ represents the peak area of oxaloacetic acid with the test substance, and A_0_ represents the peak area without inhibitors in the above equation.

The half-maximal inhibition concentration (IC_50_), a critical metric for assessing drug-induced apoptosis, was employed. IC_50_, a standard measure in pharmacology, evaluates a drug’s effective concentration. In this study, the IC_50_ value was determined using the nonlinear curve fitting function in GraphPad Prism. Solutions of the inhibitor BMS-303141 were prepared in concentrations ranging from 0.01 µM to 8 µM, corresponding to logarithmic values of −2, −1, 0, 0.301, 0.602, and 0.903, respectively. For each, 80 µL of a 500 µg/mL citric acid solution and 110 µL of Tris buffer were combined, followed by 40 µL of the designated concentration of inhibitor solution. Then, 40 µL of enzyme solution was added, and the mixture was thoroughly stirred. The reactions were incubated at 37 °C for 50 min. Post-incubation, 120 µL of the reaction mixture was combined with 480 µL of acetonitrile, shaken vigorously, and centrifuged at 1200 r/min for 10 min to clarify the solution. The clear supernatant was filtered through a 0.22 µm membrane and analyzed by UHPLC-QTOF MS. The negative control setup mirrored the previously described protocol but substituted the BMS-303141 inhibitor with an equivalent volume of solvent.

### 4.8. Identification of Herbs with Inhibitory Effects

The following experiment investigated the inhibitory effects of the natural product extracts. First, 100 mg each of powdered *Salvia miltiorrhiza*, *Rhubarb*, *Lycium barbarum*, *Platycodon grandiflorum*, and *Rosa laevigata Michx* were weighed. The herbs underwent ultrasonic extraction with a KS-5200DE sonicator (Kunshan Ultrasonic Instrument Co., Ltd., Kunshan, China). Sonication was performed at 240 W, 40 Hz, 30 °C, for 40 min. A 10 mg:1 mL of solid–liquid ratio was used for extraction, with 75% (*v*/*v*) methanol as the solvent. The extract was filtered through a 0.22-µm membrane. Subsequently, 40 µL of the extract and negative control (75% (*v*/*v*) methanol) were separately added to the enzyme reaction system. The reaction occurred in a 37 °C for 50 min. Next, 120 μL of the enzyme reaction solution was combined with 480 μL of acetonitrile and centrifuged at 1200 r/min for 10 min. A total of 200 μL of the supernatant was purified through a 0.22-µm membrane for UHPLC-QTOF MS analysis.

Samples were obtained from three regions: Rongde Ankang Clinic in Licang District, Ankang Pharmacy in Pingdu District, and Beijing Tongren Housing in Laoshan District. *Rhubarb* was extracted with water, and *Salvia miltiorrhiza* with 20% methanol. The extracts were filtered through a 0.22-µm membrane. The extract and the negative control (extraction solvent) were individually introduced into the enzyme reaction systems. Reactions were incubated at 37 °C for 50 min. Subsequently, 120 μL of the reaction solution was mixed with 480 μL acetonitrile and centrifuged at 1200 r/min for 10 min. Then, 200 μL of the supernatant was filtered through a 0.22-μm membrane for UHPLC-QTOF MS analysis.

### 4.9. Analysis of the Inhibiting Components of Rhubarb and Salvia miltiorrhiza

(1) Analysis of the inhibiting components of *Salvia miltiorrhiza*

The *Salvia miltiorrhiza* extract and negative control (water) were introduced into separate enzyme reaction systems. Reactions were incubated at 37 °C for 50 min. Subsequently, 120 μL of the enzyme reaction solution was mixed with 480 μL of acetonitrile and centrifuged at 1200 r/min for 10 min. A total of 200 μL of the supernatant was filtered through a 0.22-μm membrane for UHPLC-QTOF MS analysis. The peak area of oxaloacetic acid was compared in the enzyme reaction with and without the *Salvia miltiorrhiza* extract. The ions were analyzed with MassHunterB.04.00 software.

(2) Analysis of the inhibiting components of *Rhubarb*

Rhubarb polysaccharide samples, labeled test articles 1 and 2, were heated and dissolved in 1 mL of water. Rhubarb anthraquinone samples (test article 3–6) were dissolved in 5 mL of 50% (*v*/*v*) ethanol. Afterward, the enzyme reaction system was added with 40 µL of each rhubarb anthraquinone solution and the negative control. Reactions were incubated at 37 °C for 50 min. Subsequently, 120 μL of the enzyme reaction solution was mixed with 480 μL of acetonitrile and centrifuged at 1200 r/min for 10 min. A total of 200 μL of the supernatant was filtered through a 0.22-μm membrane for UHPLC-QTOF MS analysis.

### 4.10. Screening Inhibitors from Marine Compound Libraries

The evaluation of herbal products revealed inhibitory effects from phenols and terpenoids. A marine chemosynthetic library containing phenolics and terpenoids was then screened. The solvent of water served as the negative control. All reagent concentrations were standardized at 10 mM. Component 15 was identified as BMS-303141. Marine phenolic compounds were numbered 1–14, and terpenoid compounds 16–34 in the library. Compounds 33 and 34, identified via SciFinder due to over 90% structural similarity to isololilide, were included. The reagent and the negative control were introduced into separate enzyme reaction systems. Reactions were incubated at 37 °C for 50 min. Subsequently, 120 μL of the enzyme reaction solution was mixed with 480 μL of acetonitrile and centrifuged at 1200 r/min for 10 min. Then, 200 μL of the supernatant was filtered through a 0.22-μm membrane for UHPLC-QTOF MS analysis. Details on the compound composition and citrate lyase inhibition are provided in Appendix B.

## 5. Conclusions

In summary, the study developed a screening system specifically for citrate lyase inhibitors using UHPLC-QTOF MS. This system facilitates the targeted exploration of marine compound libraries directed by the structural attributes of herbal active ingredients. Notably, it revealed that phenolic compounds in the marine compound library showed a strong inhibitory effect on citrate lyase. The inhibitory effect of phenolic compounds exceeded 50% at a reagent concentration of 10 mM. In contrast, terpenoids exhibited variable inhibition levels. This discovery provides significant insights into marine drug usage, further affirming the efficacy of isolating bioactive components from herbal medicines and screening marine compound libraries for structural properties.

The bacterial citrate lyase exhibits almost no significant sequence or structural homology with ATP citrate lyase (ACL). The bacterial citrate lyase exists only in a few species of bacteria. This suggests that targeting it as a molecular marker likely has a minimal impact on humans. However, it is noteworthy that, despite the differences in structure and function between these two enzymes, inhibitors of bacterial citrate lyase might also inhibit ACL due to cross-reactivity. Based on this hypothesis, the next step in research will focus on animal models to evaluate the safety and efficacy of citrate lyase inhibitors in vivo, thus providing valuable experimental data for future clinical studies.

## Figures and Tables

**Figure A1:**
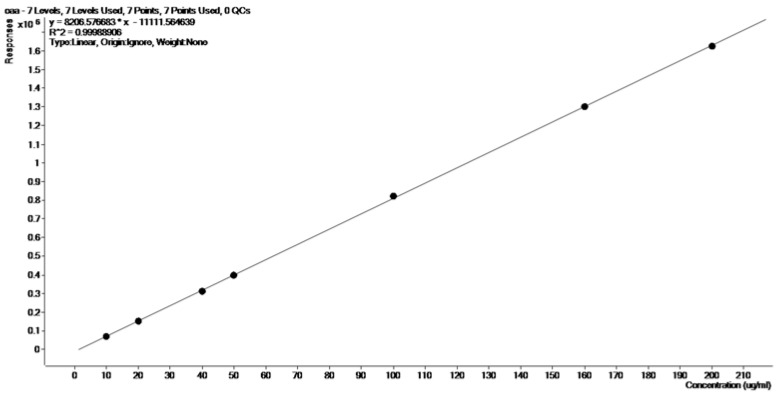
Linear plot of oxaloacetic acid.

**Figure A2:**
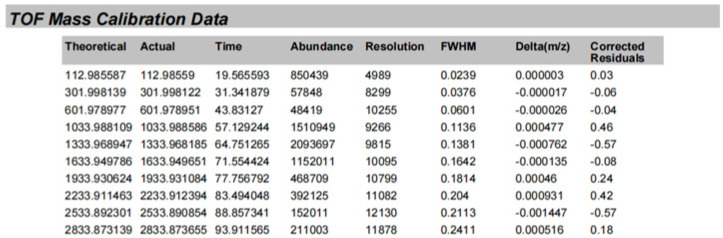
The error obtained when calibrating.

## Data Availability

Data are contained within the article; further inquiries can be directed to the corresponding author.

## Appendix A. Methodological Validation

**Table A1:** Linear regression equation and coefficient of determination of oxaloacetic acid.

Compound	Linear Range (μg/mL)	Linear	Slope (±SD)	Intercept (±SD)	R^2^
Oxaloacetic acid	10–200	y = 8306.58x − 11111.56	8206.57668 ± 38.65798	−11,111.56465 ± 4137.8749	0.9999

**Table A2:** The typical standard curves of oxaloacetic acid (*n* = 3).

Batch	Slope (±SD)	Intercept (±SD)	R^2^
1	8198.66972 ± 15.16927	−12,681.96387 ± 1640.80757	0.9998
2	9699.73101 ± 142.90583	−7440.10026 ± 15,296.36055	0.9989
3	8880.74623 ± 160.50934	33,829.95929 ± 17,180.60581	0.9984

**Table A3:** Accuracy and precision (*n* = 6).

QC Concentration (mg/L)	Intraday		Interday	
	Accuracy (RE, %)	Precision (RSD, %)	Accuracy (RE, %)	Precision (RSD, %)
10	99.44	1.75	103.44	8.45
20	96.27	2.65	98.38	7.85
50	92.72	0.96	90.63	11.23
160	97.41	1.03	95.67	6.52

**Table A4:** Stability of oxaloacetic acid in buffer solution (*n* = 6).

QC Concentration (mg/L)	Bench-Top Stability		Autosampler Stability		Freeze–Thaw Stability		Long-Term Stability	
	Accuracy (RE, %)	Precision (RSD, %)	Accuracy (RE, %)	Precision (RSD, %)	Accuracy (RE, %)	Precision (RSD, %)	Accuracy (RE, %)	Precision (RSD, %)
10	90.71	5.78	104.79	1.67	100.07	2.83	88.41	3.41
20	99.87	4.92	88.79	14.23	98.37	9.95	91.81	2.07
50	100.47	2.45	86.10	1.97	85.36	2.55	89.28	6.35
160	106.95	3.04	94.35	8.47	87.25	3.87	101.73	8.96

**Table A5:** Matrix effect of oxaloacetic acid in buffer solution (*n* = 6).

Compound	QC Concentration (mg/L)	Recovery Rate (%)	RSD (%)
Oxaloacetic acid	20	105.5	5.8
	50	103.6	7.1
	160	97.0	1.2

## Appendix B

**Table A6:** Screening results of the marine compound library.

**Phenolic Compound**	**CAS**	**Inhibition Rate**	**Inhibition Rate**	**Inhibition Rate**	**Mean**	**SD**
1	118-79-6	84.18%	83.19%	79.77%	82.38%	2.31%
2	585-76-2	57.43%	60.33%	54.99%	57.58%	2.67%
3	120-32-1	88.82%	87.44%	89.11%	88.46%	0.89%
4	608-33-3	80.80%	77.20%	81.85%	79.95%	2.44%
5	41833-13-0	46.77%	53.22%	54.09%	51.36%	4.00%
6	76045-71-1	70.90%	64.63%	72.33%	69.29%	4.10%
7	1200-93-7	50.74%	52.96%	59.71%	54.47%	4.67%
8	4526-56-1	80.06%	77.60%	78.04%	78.57%	1.31%
9	3354-82-3	90.79%	90.04%	78.95%	86.59%	6.63%
10	84743-75-9	74.74%	74.02%	78.37%	75.71%	2.33%
11	18000-24-3	72.88%	72.81%	77.45%	74.38%	2.66%
12	84743-76-0	67.71%	62.45%	75.26%	68.47%	6.44%
13	116569-08-5	69.62%	65.36%	68.84%	67.94%	2.27%
14	9005-32-7	88.65%	87.59%	87.25%	87.83%	0.73%
15	943962-47-8	67.68%	68.14%	68.08%	67.97%	0.25%
**Terpenoid Compound**	**CAS**	**Inhibition Rate**	**Inhibition Rate**	**Inhibition Rate**	**Mean**	**SD**
1	943962-47-8	67.68%	68.14%	68.08%	67.97%	0.25%
2	1117-52-8	40.13%	41.63%	51.08%	44.28%	5.94%
3	472-61-7	--	--	--	--	--
4	7235-40-7	--	--	--	--	--
5	559-74-0	0.90%	3.61%	23.44%	9.32%	12.31%
6	7683-64-9	--	--	--	--	--
7	15905-32-5	96.95%	86.25%	97.05%	93.42%	6.21%
8	57-88-5	16.31%	40.64%	34.82%	30.59%	12.70%
9	434-16-2	--	--	--	--	--
10	67-97-0	--	--	--	--	--
11	11103-57-4	14.79%	42.88%	30.62%	29.43%	14.08%
12	59-02-9	2.40%	30.57%	--	16.49%	19.92%
13	11032-49-8	--	--	--	--	--
14	84-80-0	--	--	--	--	--
15	1406-65-1	54.71%	78.18%	22.20%	51.70%	28.11%
16	68-26-8	90.66%	66.19%	27.58%	61.48%	31.80%
17	77-06-5	71.06%	71.87%	19.78%	54.24%	29.84%
18	150-86-7	--	--	--	--	--
19	17092-92-1	--	--	--	--	--
20	15356-74-8	--	--	--	--	--

**Table A7:** Structural formulae of the constituents of the marine compound library.

Phenols CAS	Structural Formulae	Terpenoids CAS	Structural Formulae
118-79-6		943962-47-8	
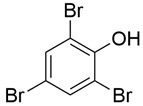
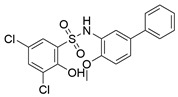
585-76-2		1117-52-8	
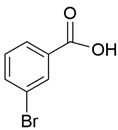
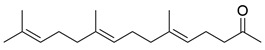
120-32-1		472-61-7	
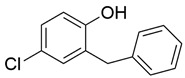
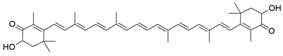
608-33-3		7235-40-7	
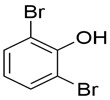
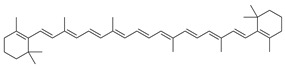
41833-13-0		559-74-0	
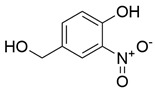
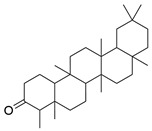
76045-71-1		7683-64-9	
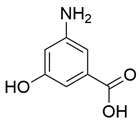
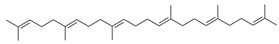
1200-93-7		15905-32-5	
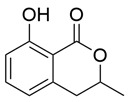
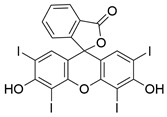
4526-56-1		57-88-5	
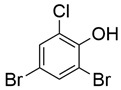
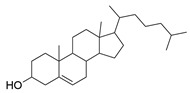
3354-82-3		434-16-2	
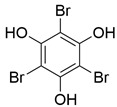
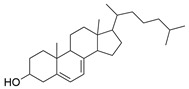
84743-75-9		67-97-0	
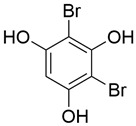
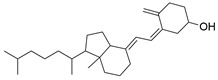
18000-24-3		11103-57-4	
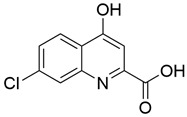
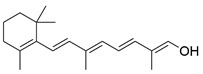
84743-76-0		59-02-9	
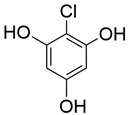
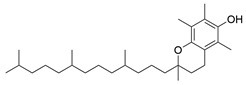
116569-08-5		11032-49-8	
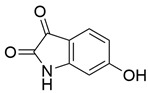
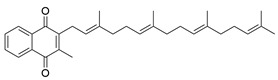
9005-32-7		84-80-0	
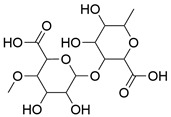
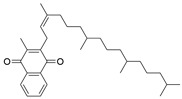
943962-47-8		1406-65-1	
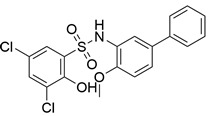
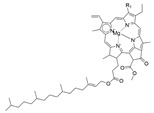
		68-26-8	
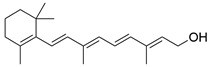
		77-06-5	
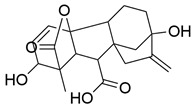
		150-86-7	
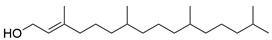
		17092-92-1	
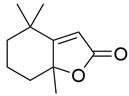
		15356-74-8	
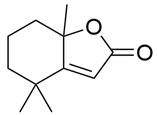

## Appendix C

**Table A8:** Purchase information from constituents of the marine compound library.

**Company**	**CAS**	**Purity (%)**	**CAS**	**Purity (%)**	**CAS**	**Purity (%)**	**CAS**	**Purity (%)**
Bide Pharmatech Ltd. (Shanghai, China)	118-79-6	98	15356-74-8	97	7683-64-9	98	76045-71-1	97
	585-76-2	99.34	1117-52-8	95	57-88-5	98	150-86-7	95
	120-32-1	98	472-61-7	95	434-16-2	95	17092-92-1	98
	608-33-3	99.86	3354-82-3	95	67-97-0	98	18000-24-3	99
	41833-13-0	99.94	84743-75-9	97	11103-57-4	350,000 IU/g	84743-76-0	97
	943962-47-8	99.15	68-26-8	98	9005-32-7	24.75		
**Company**			**CAS**	**Purity (%)**	**Company**		**CAS**	**Purity (%)**
Aladdin Global Corporation Limited			7235-40-7	96	Mclin Medical Equipment Products Co., Ltd (Shanghai, China)		15905-32-5	90
Aladdin Global Corporation Limited			559-74-0	F579523 (SKU)			59-02-9	99
Shanghai Topscience Co., Ltd. (Shanghai, China)			1200-93-7	TN7156 (SKU)			11032-49-8	98
Shanghai Topscience Co., Ltd. (Shanghai, China)			4526-56-1	TN7197 (SKU)			84-80-0	98
Shanghai Topscience Co., Ltd. (Shanghai, China)			116569-08-5	TN7147 (SKU)			1406-65-1	C832317 (SKU)
							77-06-5	90
